# When leaders “stoop”: dual-path mechanisms and social connection reconstruction of humble leader behavior to dispel occupational stigma consciousness

**DOI:** 10.3389/fpsyg.2025.1614345

**Published:** 2025-09-29

**Authors:** Kaituo Zhang, Shuo Ma, Yaqian Zhang

**Affiliations:** School of Economics and Management, Nanjing Tech University, Nanjing, China

**Keywords:** humble leader behavior, occupational stigma consciousness, social isolation, professional identification, leadership relational identification, non-decent workers

## Abstract

This study aims to explore the mechanism and path of influence of humble leader behavior on the occupational stigma consciousness of non-decent workers. Based on social exclusion theory and resource conservation theory, we constructed a dual-path model of “social isolation-professional identification.” We introduced leadership relational identification as a moderator variable to systematically analyze how humble leaders can indirectly reduce occupational stigma consciousness by reducing social isolation and enhancing professional identification, and then promote proactive behavior and job accomplishment. Through open coding and categorizing the inter-view texts of 20 nurses using grounded theory, the study refined the relationships of the core variables to form a qualitative analysis framework of the pathways of occupational stigma consciousness, and tested the hypotheses through structural equation modeling and hierarchical regression analysis using a multi-temporal tracking design with a sample of 731 nurses in eastern China. The results of the study indicate the following: Firstly, humble leader behavior exerts a significant negative influence on occupational stigma consciousness, while concurrently promoting proactive behavior and job accomplishment. Secondly, social isolation and professional identification partially mediate the relationship between humble leader behavior and occupational stigma consciousness. Thirdly, leadership relational identification positively moderates the effects of humble leader behavior on social isolation and professional identification. From a theoretical standpoint, this study proposes a dual-path intervention model of occupational stigma consciousness, which deepens the mechanism of leadership and the theory of stigma management. From a practical standpoint, it provides empirical evidence for organizations to optimize the psychological resources of non-decent workers and build inclusive environments through humble leadership behaviors. The findings of the study carry significant ramifications for the enhancement of the experience of non-decent workers and the augmentation of the sustainable performance of organizations.

## 1 Introduction

In practice, certain occupations are often stigmatized or discriminated against, leading to profoundly negative feelings among their practitioners, particularly those viewed as “dirty work.” This phenomenon is known as occupational stigma consciousness, which encompasses the negative evaluations and stereotypes that employees perceive about their professions from society at large (Ashforth et al., 2017). For example, individuals in cleaning, delivery, and food service roles- practitioners of these “dirty jobs”- are often labeled as “stigmatized, “scorned by mainstream society, gradually marginalized, and denied the respect they rightfully deserve (Meisenbach, 2010). This social stigma creates significant interpersonal pressure and adverse emotional experiences for these workers, leading to varying degrees of withdrawal and counterproductive behaviors. Given the profound impact of occupational stigma consciousness on both employees and organizations, it has emerged as a significant challenge for academics and industry professionals to investigate the pathways and mechanisms of occupational stigma consciousness.

Stigmatization, as a negative stereotype, affects the status of a group in social relations, leading to a gradual loss of social credibility and value for the group as a whole due to unfair public perception (Erving Goffman Stigma, 2009). For employees, occupational stigma consciousness functions as a negative perceived experience, imposing significant psychological burdens and triggering depression, resentment, and emotional exhaustion (Simpson et al., 2012). Practitioners are typically the first to perceive external stigma targeting their profession (Keane, 2019). From a social constructionist lens, occupational stigma emerges from the dominant group’s imposition of a dichotomous social role hierarchy through purification logic—granting moral legitimacy to “decent” occupations while framing “non-decent” ones as societal contaminants (Douglas, 1966; Petriglieri, 2011). This stigmatization operates through dual mechanisms that profoundly shape practitioners’ experiences.

However, while existing research has revealed the potential impact of leadership behaviors on the stigmatization of employees in non-decent work, there are still gaps in the exploration of specific mechanisms and leadership traits. In particular, the role of different leadership styles in this context remains to be fully elucidated. It is noteworthy that a significant number of occupations which are subject to stigma often exhibit a humble leadership style. This leadership style exerts a distinctive influence on employees experiencing stigma.

The notion of humble leader behavior, as an emerging leadership trait, has shown promise in mitigating the stigma of non-decent work employees, due to its distinctive characteristics of openness, inclusivity, and supportiveness. These leaders have been shown to intervene effectively, offering resources and facilitating cognitive restructuring, thereby reducing the occupational stigma consciousness experienced by employees. Humble leadership is a distinct leadership style characterized by the leaders’ objective self-assessment, self-knowledge, appreciation for others’ contributions, openness to feedback, and proactive leadership in modeling teachability (Owens et al., 2013). Research has demonstrated that humble leader behavior exerts a facilitating effect on employees’ constructive behavior and job performance (Nielsen et al., 2010). Nursing managers can increase workplace innovativeness by adopting an empowering leadership style that supports nurses’ self-efficacy and distributes leadership tasks (Jønsson et al., 2021).

The first mechanism is social exclusion. Occupational stigma originates from the dominant culture’s symbolic demarcation between “Decent” and “Undecent” roles (Grant and Wade-Benzoni, 2009; Douglas, 1966). That is, by reducing occupational worth to binary moral judgments, the practitioner is given a stigmatized status of “untrustworthy attributes.” In this process, the dominant group constructs cultural symbolic standards such as “clean-dirty” and “highbrow-lowbrow” based on power discourses (Link and Phelan, 2001), and labels violence against occupations that deviate from these standards (e.g., dangerous, morally controversial, physical contact work). Thereby solidifying them as “low-status identity markers” within a “definition-label-exclusion” chain. On the one hand, professional identities have been deconstructed by stigmatized labels into “othered existences” that lack legitimacy (Costas and Fleming, 2009), resulting in practitioners not being able to gain social respect and group belonging through conventional pathways; On the other hand, stigmatization triggers a chain reaction of social exclusion, plunging practitioners into a marginalized interaction dilemma with low affective connection (Meisenbach, 2010), creating a vicious cycle of professional devaluation and social status entrenchment. Ultimately, the imposition of a “spoiled identity” systematically severs practitioners from mainstream networks. Workers who are considered “non-decent” face identity toxicity, and their professional labeling triggers moral distancing among members of society. This structural exclusion subjects practitioners to a state of social death. This exclusion impedes the accumulation of social capital, systematically devalues professional dignity, and engenders disdain from mainstream social groups, leading to marginalization in social interactions (Meisenbach, 2010). Humble leaders who adopt open and inclusive attitudes and behaviors have been shown to initiate positive communication and cooperative relationships with stigmatized occupations (Dragoni, 2005; Naumann and Ehrhart, 2005). It has been demonstrated that nursing staff who experience higher levels of job satisfaction are less likely to resign (Kelly et al., 2022). These efforts have been demonstrated to effectively dismantle barriers to social isolation and cultivate a more supportive and accepting work environment for employees, thereby mitigating their perceived stigma.

Beyond social exclusion, the second mechanism is self-internalization. Resource conservation theory posits that self-perception is shaped by social roles and others’ evaluations. Occupations, as core social identities, profoundly influence psychological states. Prolonged exposure to occupational stigma triggers stigma internalization—external prejudices become internalized as negative self-schemas through cognitive assimilation, forming a vicious cycle of “occupational shame → self-devaluation → emotional depletion” (Simpson et al., 2012). The stress internalized by this stigma activates the Conservation of Resources (COR) theory (Hobfoll, 1989), induces mood disorders such as depression, and prompts practitioners to avoid careers to protect resources as much as possible. The professional identification of employees engaged in non-decent work is often severely impacted when they encounter a sense of stigma. This stigma can lead to a decline in the perception of the value and significance of one’s profession, which in turn can hinder the ability to derive a sense of fulfillment and pride from one’s work (Bonfanti et al., 2025). This, in turn, can hurt work engagement and performance (Ashforth et al., 2007; Lopina et al., 2012; Bove and Pervan, 2013; Asher, 2014; Shantz and Booth, 2014; Makkawy and Scott, 2017). Conversely, humble leaders exhibit distinctive leadership qualities, such as, They are characterized by a realization of value reaffirmation and a reconstruction of the meaning of work through objective and accurate assessment, authentic and comprehensive self-knowledge, taking the lead in modeling and openly accepting employees in terms of teachability, respecting their values and contributions, encouraging their development, etc. As articulated by Kahn (1990) and further elaborated by Li et al. (2015), this approach can facilitate a reconceptualization and affirmation of professional roles among employees, thereby enhancing their professional identification and, to a certain extent, counteracting the deleterious effects of stigma. This, in turn, can lead to enhanced work engagement and performance (Owens et al., 2013; Qin et al., 2020; Morris et al., 2005). A study of nurses in Pakistan found that nurses’ perceptions of their environment have an impact on the quality of care and job satisfaction.

Leadership relational identification is defined as the degree to which employees identify with their relationship with their leaders. It plays an important role in employees’ interpretations of and responses to leadership behaviors (Zhong et al., 2024). The psychological empowerment of employees that is engendered by humble leadership has been demonstrated to result in a more profound relationship identification (Barattucci et al., 2025). When employees exhibit a high level of relational identification with their leaders, they are more inclined to interpret their leaders’ behaviors positively and are better able to resist the effects of social isolation and professional identification challenges, even in the face of these negative factors (Bass, 1985). Research indicates that employees who possess a robust sense of organizational identity tend to exhibit elevated levels of trust and a profound sense of purpose in their work, which, in turn, fosters enhanced levels of wellbeing (Teresi et al., 2024). Specifically, concerning social isolation, employees with a high level of leadership relational identification may be more active in team activities and proactive in communicating with coworkers and leaders, thereby reducing social isolation. Conversely, employees with low leadership relational identification may be more likely to feel ostracized, thereby exacerbating social isolation, which in turn affects their sense of stigma. In terms of professional identification, employees with high leadership relational identification are more likely to receive support and recognition from their leaders for their professional development, thereby enhancing their professional identification (Xu et al., 2024). This phenomenon has been demonstrated to facilitate enhanced resilience in the face of stigma and promote heightened levels of work engagement. Conversely, employees with low leadership relational identification may experience a lack of such support, potentially leading to diminished professional identification and heightened vulnerability to the impact of stigma.

Furthermore, this study selected proactive behavior and job accomplishment as outcome variables, and introduced the positive leadership trait of humble leader behavior, as well as the mediating effects of social isolation, professional identification and the moderating variable of relational identification, to comprehensively analyze the paths of non-decent work occupational stigma consciousness and its mechanism. This study contributes to enrich the leadership theory and practice by providing a theoretical basis and practical guidance for improving the work experience of non-decent work employees. By acquiring a more profound comprehension of these mechanisms, organizations can enhance their support for non-decent work employees, promote their proactive behaviors and job accomplishment, and facilitate the sustainable development of the organization.

**Figure 1:**
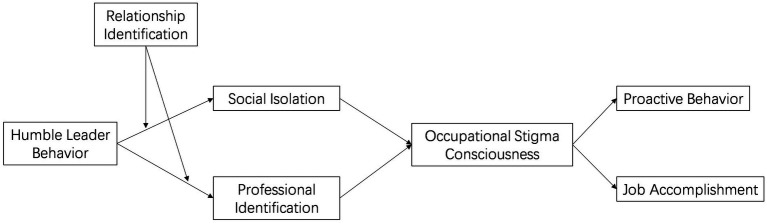
Theoretical model of the study.

The overarching model diagram for this study is depicted in Figure 1.

## 2 Literature review and hypotheses

### 2.1 The impact of humble leader behavior on proactive behavior and job accomplishment

At the beginning of the 21st century, the advent of positive psychology has precipitated an exhaustive examination of character strengths (Owens et al., 2013). Humble leader behavior is founded on the principles of psychological safety theory (Edmondson, 2004), which emphasizes the establishment of cooperative interaction networks through the weakening of power distance (e.g., transparent two-way communication, recognition of employee contributions) (Carmeli et al., 2010; May et al., 2004). A distinguishing feature of humble leaders is their consistent motivation to “learn from others” (Lawrence and Nohria, 2002), as evidenced by behaviors such as taking credit for the accomplishments of their subordinates, actively seeking feedback, and empowering decision-making processes (Burke, 2006; Lubit, 2002). This shared theoretical foundation underscores the significance of employee psychological safety and organizational effectiveness.

Humble leader behavior systematically enhances employee work attitudes through cognitive appraisal and psychological resource mechanisms, as evidenced by in-depth interviews, case studies (Owens et al., 2013; Morris et al., 2005), and large-scale surveys (Ou et al., 2014; Ou et al., 2018; Wang et al., 2018). Such leadership improves job satisfaction, organizational commitment, and task performance, partially mediated by employees’ positive perceptions of leader trustworthiness and support (Russell et al., 2004).

In Organizational Behavior, Proactive behavior is defined as a self-initiated, future-focused, and change-oriented work pattern, where employees take the initiative to identify problems and create opportunities for change rather than waiting for instructions (Frese et al., 1997). The efficacy of proactive behavior is highly contingent on interaction styles. Performance-oriented leaders may suppress subordinates from expressing initiative due to authority defensiveness (Sijbom et al., 2015), leading employees to perceive proactive behavior as resource depletion rather than value creation (Cangiano et al., 2018). In contrast, humble leaders foster psychologically safe environments by publicly crediting subordinates (Burke, 2006) and adopting team suggestions (Lubit, 2002; Carmeli et al., 2010; Edmondson, 2004), thereby enhancing employees’ sense of dignity (Kahn, 1990), work engagement (Owens et al., 2013), and proactive sustainability. Critically, the complementarity between leadership traits and employee behaviors proves pivotal: extroverted leaders tend to misinterpret proactive behavior as a dominance threat to dominance (Grant et al., 2011), whereas humble leaders build non-competitive interactions through listening, showing vulnerability, and recognition (Parker et al., 2019) to optimize team effectiveness. Based on these mechanisms, we propose:

*H1a*: Humble leader behavior has a significant positive effect on employees’ proactive behavior.

As a positive perception of one’s own occupational ability and value contribution, job accomplishment is not only a core indicator of occupational efficacy, but also a key variable in predicting burnout (Maslach et al., 2001). When the level of job accomplishment decreases, employees are prone to fall into a vicious cycle of self-efficacy deficiency, which is manifested in physical and mental exhaustion, decline in occupational competence, and lack of endogenous motivation (Maslach et al., 2001). Humble leader behavior empowers employees’ job accomplishment through multiple pathways. Grounded in social exchange theory and self-determination theory, humble leaders cultivate environments of high psychological safety and low power distance (Owens et al., 2013). Specifically, by recognizing subordinates’ contributions (Kahn, 1990) and valuing bidirectional feedback (Owens et al., 2013), these leaders amplify employees’ sense of dignity and autonomy (Tierney et al., 1999), thereby stimulating intrinsic motivation and creativity (Qin et al., 2020). For instance, humble leader behavior mitigates evaluation anxiety, enabling employees to “be themselves” (Kyriacou, 1987) while promoting responsibility and problem solving through empowering behaviors (Hsu et al., 2011). Such supportive interactions not only enhance employees’ sense of competence but also strengthen their identification with organizational goals (Duran et al., 2004), creating a virtuous cycle of achievement-driven work.

Empirical studies further substantiate humble leader behavior’s facilitative role in job accomplishment. By fostering knowledge sharing and collaboration with employees, humble leader behavior significantly enhances team effectiveness (Owens et al., 2013), while their trait of openness motivates employees to embrace innovative risks (Wang et al., 2018). Moreover, humble leader behavior’s positive associations with job satisfaction and organizational commitment, coupled with its suppression of turnover intent (Rego et al., 2019), underscore its long-term significance for sustainable job accomplishment. Crucially, the humble leader’s behavior can enhance the sense of accomplishment in individuals by providing immediate feedback. Based on these mechanisms, we propose:

*H1b*: Humble leader behavior has a significant positive effect on employees’ job accomplishment.

### 2.2 Occupational stigma consciousness

Leaders who adopt a humble approach can mitigate employees’ occupational stigma consciousness through interventions such as the provision of resources and cognitive reframing. Characterized by self-awareness, others’ value recognition, and learning-oriented modeling (Li et al., 2015), humble leaders counteract the occupational stigma effect via relational identity reconstruction. For example, Kreiner et al. (2006) found that leaders at a waste management firm reframed the stigmatized role as socially responsible by highlighting contributions of enterprises in the circular economy, reshaping employees’ professional identity. Similarly, Wang et al. (2024) demonstrated that team-building activities (e.g., family appreciation events) reduced isolation among “non-decent” jobholders and bolstered professional pride. Additionally, humble leader behavior enhances emotional well-being and alleviates work stress, fostering employees’ workplace passion and motivation in their work (Bulutlar and Başkaya, 2015; Fukui et al., 2019; Jensen and Solheim, 2020; Bratslavsky et al., 1998). Through humble leader strength spotlighting, employees amplify career efficacy and reverse self-defeating cognitions (Fukui et al., 2019). Empirical evidence indicates that humble leader behavior elevates employees’ perceptions of organizational support (e.g., flexible work policies, psychological assistance) (Gaudet and Tremblay, 2017), which enhances affective commitment and thereby stimulates organizational citizenship behaviors and craftsmanship (Jensen and Solheim, 2020). By the tenets of the Conservation of Resources (COR) theory, individuals exhibit a propensity to pursue the acquisition, maintenance, cultivation, and protection of the resources they deem to be valuable (Perko et al., 2016). When occupational stigma creates negative feelings among practitioners, they face the threat of resource loss, which triggers individual behaviors to protect resources. In order to avoid the occurrence or further expansion of resource loss, practitioners will reduce their consumption of resources in other areas. When leadership interventions disrupt the “stigma-resource depletion” pathway, practitioners reallocate psychological resources to value-driven occupational engagement. Based on these mechanisms, we propose:

*H2*: Humble leader behavior has a significant negative effect on occupational stigma consciousness.

As an interacting product of social exclusion and self-internalization, occupational stigma consciousness negatively impacts practitioners’ psychological and behavioral systems. Gurr (1970) longitudinal study revealed that occupational stigma consciousness triggers employees’ relative deprivation—a psychological imbalance stemming from perceived deficits in occupational value and social recognition relative to reference groups. This deprivation significantly diminishes work wellbeing, a positive psychological resource that enhances performance via strengthened goal commitment and in-role performance (Erez and Isen, 2002; Nouvilas-Pallejà et al., 2018). Psychological contract theory (Rousseau, 1989) posits that practitioners experiencing a breached social respect-for-labor exchange activate cognitive reappraisal mechanisms. To alleviate the effort-recognition imbalance, they reduce job inputs (Mcmurray and Ward, 2014).

Occupational stigma consciousness erodes employees’ positive behaviors through dual mechanisms: self-verification threat (Wen et al., 2022) and resource depletion (Hunter et al., 2017). Its core risk lies in professional identification erosion. Ashforth et al. (2007) longitudinal study found that chronically stigmatized practitioners adopt self-delegitimization—denying occupational value (e.g., “my work is trivial”) to sever self-professional ties. This identity crisis directly suppresses proactive behavior: practitioners’ negative evaluations brought about by professional stigma can undermine their value judgments and sense of identity about themselves and their profession. Practitioners do not utilize their profession as a means of self-evaluation, striving to disassociate themselves from their existing group and eschewing initiatives for innovation and environmental enhancement.

Dutton et al. (2010) study of ride-hailing drivers revealed that drivers avoided contributing to platform optimization initiatives to evade the “low-skilled” label, thereby suppressing proactive behavior. Unaddressed occupational stigma directly undermines practitioners’ proactive behavior. Based on these mechanisms, we propose:

*H3a*: Occupational stigma consciousness has a significant negative effect on proactive behavior.

Beyond the aforementioned resource depletion (Huang et al., 2020), occupational stigma consciousness further suppresses employees’ positive behaviors and cognitions through self-verification threat (Kusluvan et al., 2022). Steele (1997) research indicates that individuals with strong domain identity face stereotype threat, a persistent anxiety of being negatively judged or inadvertently reinforcing domain-specific stereotypes through self-referential behaviors. This anxiety depletes emotional resources, manifesting as shame-driven affective states.

Occupational stigma not only affects cognitive domains (e.g., identity threat) but also significantly increases turnover intention and burnout (Schaubroeck et al., 2018). Practitioners may face systemic social exclusion (Dutton et al., 2010), and such marginalization heightens emotional exhaustion through shame and negative emotions, reducing work engagement (Ashforth et al., 2007). Notably, high-stigma employees receive lower performance evaluations (Lai et al., 2013). Social work and other stigmatized professions are more prone to stress (Bove and Pervan, 2013; Johnson et al., 2017). The JD-R model suggests excessive job demands deplete psychological resources, while adequate support enhances engagement and fulfillment (Bakker and Demerouti, 2007; Locke et al., 2017). Social cognitive theory further indicates success expectancy is higher in low-stress states (Bandura, 1997). Based on these mechanisms, we propose:

*H3b*: Occupational stigma consciousness has a significant negative effect on job accomplishment.

### 2.3 Social isolation

The social environment exerts a profound influence on individuals’ health and psychological wellbeing. When employees experience chronic deficiencies in social support and interaction, they become marginalized within relational networks, a condition termed social isolation (Beukering et al., 2022). In the field of organizational management, leadership style has a significant impact on employees’ social isolation. Humble leader behavior, as a “bottom-up” leadership style, emphasizes the leader’s ability to build equalized hierarchical relationships through self-awareness, openness to listening, and respect for employees’ values. At the heart of this leadership style is the leader’s respect and trust in employees, which creates a positive organizational climate. Humble leader behavior can effectively alleviate social isolation in the workplace by enhancing employees’ sense of organizational identity and psychological security (Gaudet and Tremblay, 2017; Benz et al., 2019). Specifically, humble leader behaviors have been shown to enhance employees’ sense of self-worth at the organizational level and reduce communication barriers due to power gaps. These conditions have the potential to reduce social exclusion within the team as a whole (Farh et al., 2007). This type of leader behavior has been shown to improve employees’ psychological state, as well as to increase teamwork and organizational effectiveness.

Humble leader behaviors are positively correlated with the quality of leader-employee exchanges (Atwater and Carmeli, 2009). By fostering pro-active behaviors and enhancing job fulfillment, humble leaders can assist employees in mitigating social exclusion, thereby elevating their organizational status (Xu et al., 2024). The leadership style and organizational environment have been demonstrated to exert a significant influence on the extent of employees’ social isolation. A seminal experiment on the compensatory effect of competence demonstrated that high-quality task performance by members of a disadvantaged group can transcend power constraints and reconfigure the group’s status, thereby effectively reducing the group’s social isolation (Johnston and Ricciardelli, 2022). However, deficiencies in occupational culture can undermine social identity formation and hinder individual employees’ ability to mitigate isolation through shared narratives or group belonging (Bentein et al., 2017). Based on these mechanisms, we propose:

*H4a*: Humble leader behavior has a significant negative effect on social isolation.

Stigmatization is defined as the “lowering of an individual’s social identity” (Erving Goffman Stigma, 2009). Occupational stigmatization can be conceptualized as a form of socio-cognitive violence that operates by linking occupational attributes to social values, thereby placing particular occupational groups at a double disadvantage in terms of their moral and social status through stigmatizing depictions (Crocker and Major, 1989). Practitioners of these occupations face not only geographic segregation, with workplaces separated from residential areas, but also experience social isolation due to the stigmatization associated with their occupations (Guerrero and Bentein, 2020). This stigma engenders social exclusion, systematically excluding practitioners from social groups and denying them access to popular emotional support and identity (Lucas and Phelan, 2012). Notably, practitioners’ perceived social isolation can lead to a deterioration in their psychological resilience and a progressive internalization of stigma, where individuals come to accept occupational stigma as a given. This dynamic perpetuates a vicious cycle of social isolation, influencing the perception of occupational stigma (Bentein et al., 2017).

For instance, sanitation workers are often stigmatized as “urban nuisance makers” rather than recognized as “environmental defenders,” leading to a decline in support-seeking behaviors among practitioners. This phenomenon is attributed to the desire to avoid secondary victimization by society (Epstein, 2007). This self-imposed social withdrawal can intensify feelings of alienation and hinder practitioners’ capacity to reclaim a sense of purpose in their profession. A longitudinal study of abortion providers demonstrated that under persistent moral condemnation and workplace harassment, practitioners developed defensive silences and consciously avoided social interactions, a pattern of behavior that ultimately led to the dual psychological distress of social isolation and chronic burnout (O’Donnell et al., 2011). Moreover, the phenomenon of stigma-labelling resulted in a perpetual state of identity anxiety among practitioners, leading to the operationalization of self-fulfilling prophecy mechanisms. Empirical studies on non-decent work practitioners reveal prevalent defensive self-regulation in social interactions, manifested through occupational reference avoidance and communicative hypervigilance. This sustained anxiety systematically reinforces social avoidance patterns, thereby exacerbating isolation (Abel, 2011). Based on these mechanisms, we propose:

*H4b*: Social isolation has a significant positive effect on occupational stigma consciousness.

Humble leader behavior as an effective leadership style can alleviate social isolation (Bentein et al., 2017) through enhancing employees’ sense of psychological security and organizational identity (Gaudet and Tremblay, 2017). This style of leadership emphasizes leaders’ respect and trust in employees, and helps practitioners overcome isolation by establishing egalitarian hierarchical relationships, thus achieving “neutralization.” Humble leaders help employees construct an “anti-stigma social network” (Hudson and Okhuysen, 2009).

The negative impact of social isolation on “dirty work” practitioners is significant. Research has shown that a lack of social and emotional interaction at work, along with perceived social isolation, is positively associated with emotional exhaustion and negatively associated with work engagement (Marshall et al., 2007). This sense of isolation prevents practitioners from developing defense mechanisms to counteract occupational stigma. For example, when individuals feel connected to a group, they are more likely to mitigate stigma-related harm through group belonging (Bentein et al., 2017). However, isolated individuals find this defense mechanism harder to achieve, leading to heightened occupational stigma consciousness. Based on these mechanisms, we propose:

*H4*: Humble leader behavior reduces occupational stigma consciousness by influencing employees’ social isolation.

### 2.4 Professional identification

Occupation is a central element in an individual’s sense of self-definition and existential value (Van Dick and Kerschreiter, 2016; Lai et al., 2013; Deery et al., 2019). Professional identification refers to the extent to which individuals internalize professional capacity as a positive self-definition (Ashforth et al., 2013), encompassing both the knowledge of belonging to an occupational group and the significance of emotions and values derived from group membership (Turner and Tajfel, 1986).

First, humble leaders objectively evaluate themselves, proactively acknowledge limitations (e.g., knowledge gaps or decision-making errors), and seek employee feedback through open communication (Owens et al., 2013). In the Humble Leader Behavior Scale, the “correct self-perception” dimension (e.g., “facing limitations and addressing them”) enhances employees’ perception of leadership credibility, thereby strengthening their recognition of professional value (Morris et al., 2005). Second, humble leaders reconstruct occupational symbolic meaning by publicly affirming the social contributions of non-decent work and emphasizing employees’ value (Ashforth and Kreiner, 1999). Conservation of Resources Theory (Hobfoll, 1989) suggests that value reaffirmation and leader recognition provide psychological resources (e.g., occupational dignity) to bolster professional identification. Finally, humble leaders reduce power distance’s inhibitory effects through organizational culture empowerment, such as establishing flat communication mechanisms (Farh et al., 2007). For instance, under humble leadership, R&D personnel’s creativity increases as they reconstruct their identity as “technical experts” rather than “low-end executives” (Nielsen et al., 2010). Based on these mechanisms, we propose:

*H5a*: Humble leader behavior has a significant positive effect on professional identification.

The stigmatization of non-decent occupations is the result of tensions at both the individual and societal levels. On the one hand, practitioners encounter self-worth denial through the application of stigmatizing labels (e.g., symbolic violence such as “inferior” and “immoral”) (Link and Phelan, 2001). On the other hand, the occupation’s social function and individuals’ survival needs give rise to intrinsic value conflicts (Link and Phelan, 2001). According to the paradoxical professional identity theory (Kreiner et al., 2006; Van Dick and Kerschreiter, 2016), this tension can lead to conflict experiences, where practitioners find themselves in a state of oscillation between “professional value affirmation” and “stigma pressure avoidance.” Professional identification, however, functions as a dynamic equilibrium mechanism that facilitates the reconfiguration of stigma coping strategies, thereby reducing practitioners’ sense of non-decent occupational stigma consciousness.

The phenomenon of professional identification has been demonstrated to play a pivotal role in mitigating occupational stigma consciousness among non-decent workers. This mitigating effect occurs through three distinct pathways: the deconstruction of stigmatizing labels, the enhancement of group affiliation, and the facilitation of resource compensation. When individuals exhibit robust professional identification, they often redefine non-decent work with positive symbolism, such as “maintenance of social infrastructure” (Roca, 2010), thereby neutralizing stigmatizing labels. For instance, practitioners with high professional identification have been shown to significantly reduce public occupational rejection by constructing identity narratives, such as “life ritualist” (Gurr, 1970). Concurrently, professional identification fosters anti-stigma coalitions (Tracy and Scott, 2006) by reinforcing group cohesion and fortifying collective psychological defenses against external stigma (Bentein et al., 2017). Compared to peers, highly identified individuals better leverage organizational resources and social capital to combat stigma pressures (Hudson, 2008). A case study by Costas and Fleming (2009) demonstrates how manufacturing skilled workers’ “skill authority” capital, accumulated through professional identification, effectively counteracts the “low education” stigma.

Non-decent work stigma induces professional identification crises and significant stress, leading to physical/mental health issues that heighten non-decent occupational stigma consciousness and organizational inefficiencies (Kitay and Wright, 2007). In call center research, occupational stigma consciousness showed a significant negative correlation with professional identification (Shantz and Booth, 2014). Given non-decent work’s frequent association with immorality and low status, practitioners experience high occupational disapproval (Lai et al., 2013; Schaubroeck et al., 2018). Nevertheless, these practitioners view their careers as crucial status markers and seek social recognition of their work’s value. Based on these mechanisms, we propose:

*H5b*: Professional identification has a significant negative effect on occupational stigma consciousness.

Based on the above analysis, humble leaders can reconstruct practitioners’ identity generation mechanism through reshaping the organizational cultural ecology in a two-way intervention of flattening the power structure and reinterpreting professional values. Specifically, by dissolving the oppression of power distance under the hierarchical system (Farh et al., 2007), strengthening the role of occupational culture in the construction of social identity, and facilitating practitioners to enhance employees’ career affirmation through shared narratives or group affiliation, creating a high level of professional identification and mitigating stigmatizing harm (Bentein et al., 2017). Professional identification reduces the psychological stress of occupational stigma consciousness by reinforcing group cohesion so that members of the stigmatized group can cope with the identity threat by getting closer to or identifying more with their group (Fan and Wang, 2024).

This composite leadership style is of special significance to the reconstruction of the professional identification of non-decent work practitioners, who have long been torn between professional stigmatizing labels and internal value demands, and whose professional identification is in urgent need of external intervention to reconcile cognitive conflicts. The humble leader’s behavior achieves this through systematic interventions that deconstruct stigmatizing labels, enhance group belonging, and compensate for resources to reshape the professional identification of non-decent work practitioners. Based on these mechanisms, we propose:

*H5*: Humble leader behavior reduces occupational stigma consciousness by influencing employees' professional identification.

### 2.5 Relational identification

Humble leader behaviors are effective in reducing subordinates’ isolation-induced negative psychological states through openness to feedback and proactive empowerment (Owens et al., 2013; Bass, 1985). However, the strength of this effect is dependent on subordinates’ leadership relational identification: when employees have a high level of employee relational identification, they perceive the leader’s support as “within the scope of responsibility” (rather than occasional extra help) (Sluss and Ashforth, 2007), which enhances the role of humble leader behaviors in reducing employee social isolation consciousness. In other words, employee relational identification serves as the key psychological bond that strengthens the efficacy of humble leader behaviors in mitigating social isolation consciousness (Zhong et al., 2024).

Subordinates with high leadership relational identification not only increase their self-efficacy through deeper interactions with their leaders (e.g., resource acquisition, skill learning) (Latham et al., 1994), but also build trust in their leaders’ roles (Kark and Shamir, 2002). This trust translates into a “social buffer” (Ashforth et al., 2007), which makes subordinates more likely to perceive emotional support and belonging security from their leaders and more motivated to work when facing isolation risks (Judge et al., 2017). Meanwhile, employee relational identification prompts subordinates to internalize team norms conveyed by the leader (Sluss et al., 2012), further reducing the tendency of social isolation through role identity reinforcement.

In situations of low employee relational identification, humble leader behaviors may be interpreted as “formalized caring” or “power tactics” (Rego et al., 2019), having a limited effect on alleviating social isolation consciousness. Conversely, high leader-subordinate relational identification leads subordinates to bind humble leader behaviors to their role values (Van Knippenberg, 2000), forming a “resist isolation together” psychological contract (Cooper and Thatcher, 2010). This moderating effect highlights the boundary role of employee relational identification in leadership effectiveness: only when it is sufficiently strong can humble leader behaviors become effective interveners against organizational social isolation (Miao et al., 2013). Based on these mechanisms, we propose:

*H6a*: Leadership relational identification moderates the negative relation between humble leader behavior and social isolation.

Professional identification fundamentally involves the internalization of occupational identity as a core component of self-definition, through which individuals derive emotional significance and value meaning from their affiliation with professional collectives (Ashforth et al., 2013). Humble leadership establishes a “reference template” for subordinate professional identity construction through sustained behavioral exemplification, including demonstrated professional ethics and emphasized work value (Bass, 1985). When employee leadership relational identification is elevated, this modeling effect becomes amplified: subordinates not only acknowledge the leader’s professional competence but also perceive them as personifications of occupational ideals (Kark and Shamir, 2002), thereby actively integrating professional norms (e.g., perfectionism, social service) into their self-concept (Jie et al., 2014). For instance, leaders who practice active listening and subordinate empowerment (Owens et al., 2013) effectively communicate the value that “professional development requires collaborative advancement.” This approach facilitates subordinates’ transition from passive executors to active co-constructors of professional identity, ultimately achieving alignment between personal objectives and occupational requirements (Shuhua and Guangwen, 2012).

The deepening of professional identification inherently relies on emotional attachment to occupational groups (Turner and Tajfel, 1986). Humble leader behavior activates this attachment through two behavioral mechanisms: first, by providing personalized support (e.g., resource allocation, career dilemma resolution) (Shin and Zhou, 2003), which fosters subordinates’ perceptions of security and belonging in professional development; second, by articulating shared visions (e.g., “our work drives industry progress”) (Bass, 1985), thereby elevating occupational goals into collective missions. Within this process, subordinates with high relational identification perceive leaders as “personified symbols” of their professional community (Hogg, 2001), transferring emotional trust in leadership to their entire occupational identity (Trybou et al., 2014). This transference intensifies subordinates’ endorsement of their professional group’s value systems (e.g., specialisation ethics) (Loi et al., 2004), while motivating proactive behaviors (e.g., innovation, accountability) (Garcia-Falières and Herrbach, 2015) to reciprocate the meaning derived from occupational identity, ultimately forming an integrated “affection-identification-action” cycle.

Professional identification is not a static outcome, but a dynamic process reinforced by continuous interaction (Ashforth et al., 2013). When humble leader behavior establishes robust connections with subordinates exhibiting high relational identification, both parties engage in a virtuous “identification-performance-feedback” cycle: subordinates demonstrate heightened work engagement and performance due to enhanced professional identification (Judge et al., 2017), while leaders reinforce occupational belongingness through recognition and empowerment (e.g., promotion opportunities, decision-making participation) (Zhong et al., 2024). For instance, a subordinate who completes critical projects with leadership support experiences strengthened professional confidence and identification, which subsequently motivates leaders to allocate additional resources for advancing higher occupational goals. This cycle demonstrates that employees’ relational identification is both the starting point for humble leaders to shape their professional identification and the cornerstone for sustaining their long-term efficacy (Miao et al., 2013; Zhang et al., 2016; Mayer et al., 2010). Based on these mechanisms, we propose:

*H6b*: Leadership relational identification moderates the positive relation between humble leader behavior and professional identification.

## 3 Materials and methods

### 3.1 Sample and procedures

In this paper, we have prepared a questionnaire based on in-depth interviews with a group of nurses. We then proceeded to analyze the questionnaire to test the hypotheses and draw conclusions. The study was conducted in Eastern China in late 2024 and consisted of one-on-one in-depth interviews with a total of 16 nurses. The respondents were selected as far as possible from young and middle-aged individuals with active minds and rich information. The interviews were semi-structured and centered on the following questions: “Why do you choose to work in this job?” “Do outsiders look at your job through colored glasses?” and “Factors affecting your feelings about your job.” Following the acquisition of the interviewees’ consent, audio recordings of the interviews were obtained, the recorded information was meticulously organized, and the interview notes and memos were completed. The total word count of the interview transcripts was 36,000. Prior to the commencement of the interviews, a comprehensive review of extant literature and measurement indicators was conducted. Following extensive discussions and a thorough verification process, a preliminary questionnaire was developed. During the interviews, a preliminary survey was conducted 180 participants from the hospitals where the interview nurses were employed. This survey was utilized for the purpose of verifying the clarity and relevance of the questionnaire items. The preliminary survey data was utilized to conduct exploratory factor analysis, as outlined in Supplementary material 1.

The text data was analyzed using NVivo 15.0 for open coding and management. Nvivo, a widely used qualitative data analysis software, is specifically designed for handling unstructured data. The textual data were initially open-coded, and since the number of initial concepts is very heterogeneous, and there is a certain degree of crossover, and the category is a reclassified combination of concepts, we further categorized the initial concepts we obtained. For categorization, initial concepts with fewer than three repetitions were excluded, and those with more than three repetitions were selected. Furthermore, we also eliminated individual inconsistent initial concepts. The utilization of Nvivo’s “Node Classification” and “Matrix Coding Query” functions is paramount. Subsequent to this, the categories were categorized according to their interrelationships and logical order at the conceptual level. This process resulted in the identification of six primary categories. The identification of the core category, “the path of professional stigma and its mechanism of action,” was accompanied by the formulation of a “storyline” that elucidates its implications. The narrative unfolds by positing that the humility exhibited by leaders can exert an indirect influence on the organizational identity and sense of belonging experienced by non-decent workers. This, in turn, is hypothesized to lead to a reduction in their sense of social exclusion and professional stigma, thereby promoting positive work that is rewarding to the organization. Concurrently, employees’ relational identification with their leaders exerts influence on the effect of humble leaders’ interventions on non-decent workers’ social isolation, amplifying their positive contribution and thus reinforcing the aforementioned impact path. Nvivo 15.0 enhances analytical depth and research validity through systematic organization, pattern recognition, and relationship mining.

We designed an online questionnaire survey, and the technical support was provided by Questionnaire Star. The survey was distributed via network publicity and promotion, and volunteers were commissioned in each region to distribute it to nurses in each regional hospital. The researchers described the purpose and requirements of the research to the nurse group, and they followed the principle of confidentiality and voluntary participation. Ultimately, 829 nurses participated in the study, and each nurse was asked to find the head of the nurses on their own to fill in the appropriate questionnaire. The data collection was completed. A total of 829 official questionnaires were distributed in this research, and finally, 764 valid questionnaires were obtained, with a recovery rate of 92.16%. After excluding the questionnaires that did not meet the inclusion criteria, the total number of valid questionnaires was 731, resulting in an overall valid recovery rate of 88.18% for the study.

The demographic information of the sample is as follows: Of the 731 nurses, 97.1% were female and 2.9% were male in terms of gender; in terms of age, 26.0% of the nurses were 25 years old and below, 20.0% were between 26 and 35 years old, 24.1% were between 36 and 45 years of age, 20.9% were between 46 and 55 years of age, and 9.0% were 55 years of age and above. Regarding educational attainment, 25.3% of nurses have received junior high school education or below, 22.7% have received high school or junior college education, 24.4% have received college education, and 27.6% have obtained a bachelor’s degree or higher. In terms of years of working experience, 20.9% of nurses have been employed for less than or equal to 3 years, 24.9% have been employed for 3–5 years, 27.8% have been employed for 5–10 years, and 26.4% have been employed for more than 10 years.

Given the temporal discrepancy between variables, this study employed the three-time-point method to collect data. The four digits at the end of the respondents’ cell phone numbers served as the sole indicator for the pre- and post-questionnaire matching. A time-separated research design approach was employed to effectively mitigate common method bias. The study’s design involved the administration of three questionnaires, with three survey administrations occurring at half-month intervals. This approach was implemented to disperse situational interference, weaken temporary emotional effects, and reduce memory and consistency motives. This strategy effectively mitigated common methodological biases, enhancing the quality of the data and the validity of the research. In this study, nurses were asked to rate a variety of factors relevant to the research (Podsakoff et al., 2003; Lin et al., 2017). First, they evaluated the humble leader behavior of their leaders and their leadership relational identification. They also provided information about their demographic characteristics. Next, they assessed their levels of social isolation and professional identification. Finally, they provided data on the occupational stigma consciousness they experienced in their work. In addition to these evaluations, nurse leaders were asked to rate the proactive behavior and job accomplishment of their nurses.

### 3.2 Measurements

Given that all of the scales utilized were initially developed in English but were employed in a Chinese context, a meticulous “double-blind translation” procedure was employed to ensure the accuracy and clarity of the scale entries (Brislin, 1980). Subjects were instructed to rate each item on a 5-point Likert scale (1 for strongly disagree, 5 for strongly agree).

The measurement of humble leader behavior was conducted using a 9-item scale developed by Owens et al. (2013). The scale’s items included inquiries such as “My leader habitually seeks feedback from others, even when it is critical.” The internal consistency coefficient for the scale was 0.935.

Leadership relational identification was measured using the 10-item scale developed by Walumbwa and Hartnell (2011). The scale included questions such as “When someone criticizes my supervisor, it feels like an insult to me.” The internal consistency coefficient of the scale was 0.936.

Social isolation was measured using the Friendship Scale, a tool developed by Hawthorne (2006). This scale involved reversing items 1, 3, and 4 and then summing all items to create a score. Scores ranged from 0 to 24, with high scores denoting social connectedness and 0 representing social isolation. The scale incorporated questions such as “In the past 4 weeks, it has been easy to tolerate others.” The internal consistency coefficient of the scale was determined to be 0.934.

Professional Identification was measured using a modified 3-item scale by Wu et al. (2024) that reflects occupational identity (nurses) rather than organizational identity, with scale questions such as, “Over the last month, when someone criticizes the nursing profession, it feels like a personal insult.” The internal consistency coefficient for the scale was 0.870.

Occupational stigma consciousness was measured using a 10-item scale developed by Pinel and Paulin (2005) with questions such as “Stereotypes about women have not affected me personally.” The internal consistency coefficient of the scale was determined to be 0.959, indicating a high degree of internal reliability.

Proactive behavior, utilizing nine of the 27 items Griffin et al. (2007) developed to assess performance, encompasses three dimensions: individual task pro-activity, team member proactivity, and organization member proactivity. Examples of statements include, “Initiated better ways of doing your core tasks.” The scale demonstrated an internal consistency coefficient of 0.954, indicating its reliability and validity.

To assess work achievement, the Chinese Maslach Burnout Inventory (CMBI), developed by was employed. This inventory comprises three dimensions: depletion, depersonalization, and diminished sense of accomplishment. The measurement of work achievement was determined by five items, including inquiries such as “This nurse can effectively solve patients’ problems.” The internal consistency coefficient of the scale was 0.921.

The following control variables were employed: By the research designs of Cho et al. (2021) and Owens et al. (2013), employees’ gender, age, education, and years of experience were utilized as control variables. Specifically, gender was coded as “1” for males and “2” for females. Age was coded as “25 years and below” was coded as “1,” “26–35 years old” was coded as “2,” “36–45 years old” was coded as “3,” “46–55 years old” was coded as “4,” and “55 and over” was coded as “5.” Regarding educational attainment, junior high school and below is coded as “1,” high school or junior college is cod-ed as “2,” college degree is coded as “3,” and bachelor’s degree or above is coded as “4.” Concerning years of work experience, less than or equal to 3 years is coded as “1,” three to 5 years is coded as “2,” five to 10 years is coded as “3,” and more than 10 years is coded as “4.”

## 4 Results

### 4.1 Validation factor analysis

**Figure 2:**
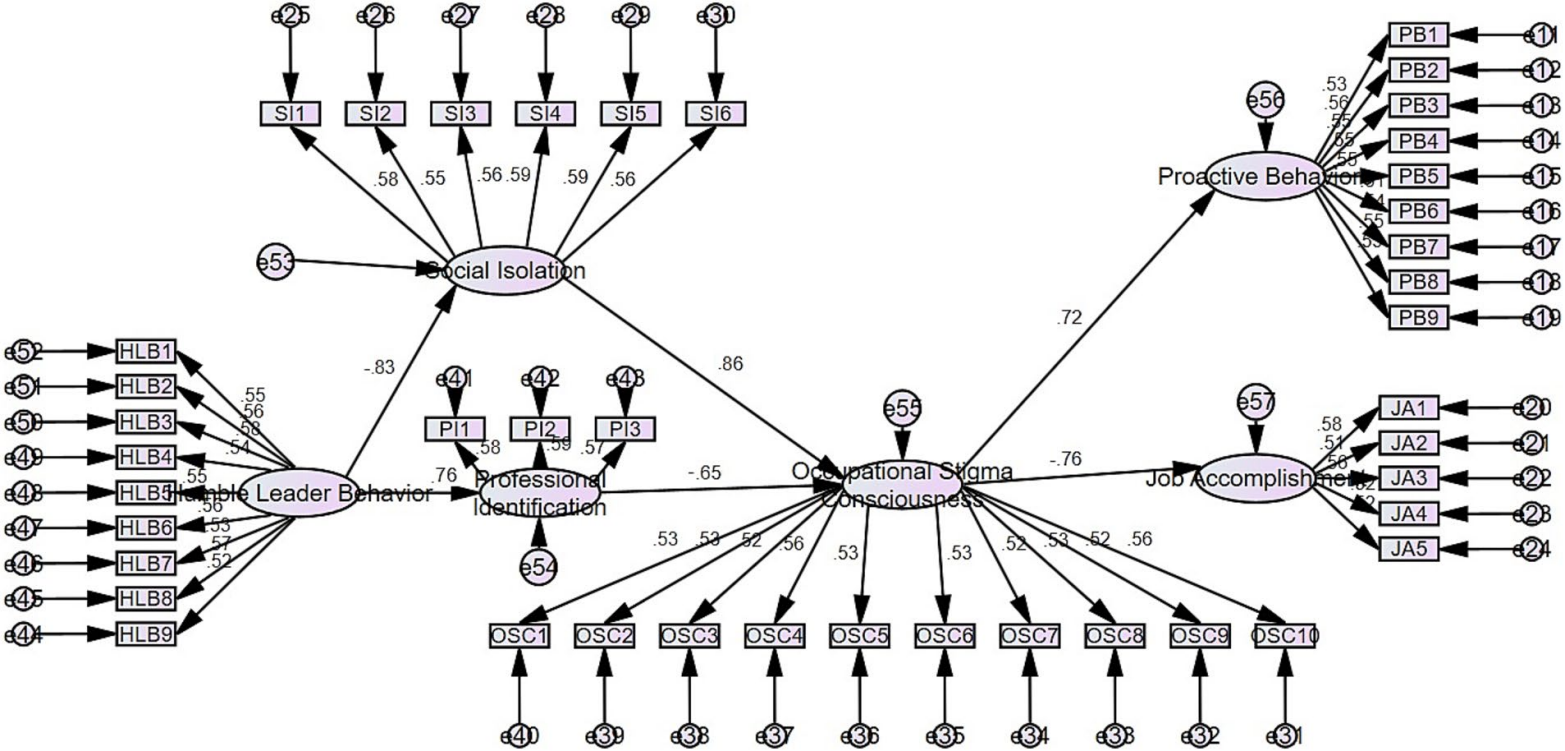
Overall relationship model.

To assess the discriminant validity of the measured variables, we employed a confirmatory factor analysis. As illustrated in Table 1, in comparison with the six-factor model (job accomplishment and proactive behaviors combined as one factor), the five-factor model (job accomplishment and proactive behaviors combined as one factor, social isolation and professional identification combined as one factor), the four-factor model (job accomplishment and proactive behaviors combined as one factor, social isolation and professional identification combined as one factor, humble leader behavior and relationship identification combined as one factor), the three-factor model (occupational stigma consciousness, job accomplishment, and proactive behaviors combined as 1 factor, social isolation and professional identification combined as 1 factor, humble leader behavior and relational identification combined as one factor), the two-factor model (occupational stigma consciousness, job accomplishment, and proactive behaviors combined as 1 factor, humble leader behavior, relational identification, social isolation, and professional identification combined as one factor), and the one-factor model (all variables combined into one factor), the seven-factor (occupational stigma consciousness, job accomplishment, proactive behavior, humble leader behavior, relation-al identification, social isolation, and professional identification) model demonstrated optimal fit validity. The model’s goodness of fit was substantiated by the following in-dices: a chi-square value of 2,513, degrees of freedom (df) of 1,253, a comparative fit index (CFI) of 0.8, a non-normed fit index (NNFI) of 0.882, a root mean square error of approximation (RMSEA) of 0.037, and a root mean square residual (RMR) of 0.067. These findings collectively indicate that the measurement variables possess adequate discriminant validity. The AMOS program was utilized in the construction and evaluation of the factors influencing occupational stigma consciousness and the path model, as illustrated in Figure 2.

**Table 1:** Results of validation factor analysis.

Model	$\chi^{2}/\mathrm{df}$	$\chi^{2}$	df	CFI	NNFI	RMSEA	RMR
One-factor model	5.257	9584.658	1823.218	0.367	0.513	0.159	0.156
Two-factor model	4.998	7969.243	1594.486	0.425	0.526	0.135	0.114
Three-factor model	4.356	6856.254	1573.979	0.534	0.535	0.116	0.123
Four-factor model	3.985	5986.256	1502.197	0.610	0.586	0.059	0.098
Five-factor model	3.259	4635.253	1422.293	0.648	0.649	0.053	0.079
Six-factor model	3.214	4126.579	1283.939	0.735	0.723	0.048	0.075
Seven-factor model	2.006	2513.694	1253.088	0.800	0.882	0.037	0.067

The present study employed the SPSS26.0 software to assess the reliability and validity of the scientific scales utilized in this research. These scales were assessed using Cronbach’s *α*, average variance extracted (AVE), composite reliability (CR), KMO, and Bartlett’s test. As demonstrated in Table 2, the Cronbach’s α values for all variables ranged from 0.723 to 0.841, thus exceeding the threshold of 0.7. The AVE values ranged from 0.417 to 0.637, and the CR values ranged from 0.604 to 0.810. Despite the fact that the AVE values for certain variables were below 0.5, as indicated by the standards established, the AVE values were all above 0.4, and the CR values were all above 0.6. This finding suggests that the convergent validity and structural validity of the questionnaire met the restrictive level. As demonstrated in Table 3, the KMO value of 0.879 is greater than 0.8, and the Bartlett test significance is less than 0.001. In summary, the scientific scale utilized in this study exhibited commendable reliability and validity.

**Table 2:** Reliability and validity tests.

Variable	Cronbach’s α	AVE	CR
Relationship identification	0.836	0.599	0.810
Humble leader behavior	0.735	0.417	0.806
Social Isolation	0.734	0.528	0.745
Professional identification	0.770	0.637	0.604
Occupational stigma consciousness	0.841	0.596	0.808
Job accomplishment	0.754	0.491	0.672
Proactive behavior	0.723	0.595	0.790

**Table 3:** KMO and Bartlett tests.

KMO test		0.879
Bartlett Sphericity test	Approximate chi-square	648.078
	Degree of freedom	21
	Significance	0.000

### 4.2 Descriptive statistics analysis

Table 4 presents a summary of the mean, standard deviation, and correlations of the primary and control variables of the present study. The results of the descriptive statistical analysis indicated a significant correlation between humble leader behavior, occupational stigma consciousness, social isolation, and occupational identification. Specifically, humble leader behavior exhibited a significant negative correlation with occupational stigma consciousness (*r* = −0.741, *p* < 0.01) and a significant negative correlation with social isolation (*r* = −0.735, *p* < 0.01), and a significant positive correlation with occupational identification (*r* = 0.661, *p* < 0.01). Occupational stigma consciousness demonstrated a significant negative correlation with proactive behavior (*r* = −0.649, *p* < 0.01) and a significant negative correlation with job accomplishment (*r* = −0.733, *p* < 0.01). These findings serve to establish a preliminary relationship between the two variables and provide initial data support for the hypothesis of this study. To ascertain the causal relationship of the variables in the research hypothesis, a hierarchical regression analysis was conducted.

**Table 4:** Means, standard deviations and correlation coefficients of variables.

Items	Variables	Mean	SD	2	3	4	5	6	7	8	9	10	11
1	Gender	1.971	0.167										
2	Age	2.67	1.305	0.515									
3	Education attainment	2.543	1.144	0.047	0.519								
4	Working hours	2.596	1.09	0.006	−0.023	0.56							
5	Relational identification	2.973	0.804	−0.014	0.041	−0.043	0.547						
6	Humble leader behavior	3.012	0.842	−0.013	0.009	0.05	0.358***	0.563					
7	Social isolation	2.986	0.883	0.022	0.028	−0.062*	−0.249***	−0.735***	0.573				
8	Professional identification	2.921	0.991	−0.078**	−0.009	0.038	0.205***	0.661***	−0.704***	0.581			
9	Occupational stigma consciousness	2.951	0.81	0.017	0.007	−0.067*	−0.205***	−0.741***	0.759***	−0.678***	0.544		
10	Job accomplishment	2.95	0.872	−0.019	−0.021	0.013	0.149***	0.669***	−0.658***	0.548***	−0.649***	0.539	
11	Proactive behavior	2.977	0.818	−0.009	0.007	0.059*	0.154***	0.719***	−0.721***	0.649***	−0.733***	0.745***	0.543

*n* = 731,***, **, * represent 1, 5, and 10% significance levels, respectively.

### 4.3 Hypothesis testing

In order to verify the proposed hypothesis, the present study implemented a stratified regression analysis. The results of the analysis are presented in Table 5. Hypothesis 1a predicted that humble leader behavior has a significant positive effect on proactive behavior. As shown in Table 5, adding only the control variable in Model 1 and further adding the independent variable humble leader behavior in Model 2 showed that humble leader behavior has a significant positive effect on proactive behavior (*β* = 0.718, *p* < 0.001), and Hypothesis 1a was supported.

**Table 5:** Hierarchical regression results (*N* = 731).

Variables	Model					
	Proactive behavior			Job accomplishment		
	1	2	3	4	5	6
Gender	0.008	−0.001	0.030	0.016	0.010	0.032
Age	−0.010	0.001	0.003	−0.019	−0.010	−0.008
Education attainment	0.008	0.001	0.004	−0.022	−0.028	−0.026
Working hours	0.060*	0.024	0.011	0.013	−0.021	−0.030
Humble leader behavior		0.718***	0.387***		0.670***	0.414***
Occupational stigma consciousness			−0.448**			−0.346***
$R^{2}$	0.004	0.518	0.604	0.001	0.449	0.502
$\Delta R^{2}$	−0.002	0.514	0.604	−0.004	0.445	0.498
*F*	0.682	155.646**	186.535**	0.219	118.057**	121771**

Variables	Model				
	Occupational stigma consciousness				
	7	8	9	10	11
Gender	0.058	0.064**	0.064**	0.044*	0.048*
Age	0.016	0.005	−0.018	0.001	−0.012
Education attainment	0.000	0.007	0.003	−0.007	−0.006
Working hours	−0.065	−0.027	−0.025	−0.017	−0.018
Humble leader behavior		−0.740***	−0.518***	−0.402***	−0.342***
Professional identification			−0.335***		−0.192***
Social isolation				0.461***	0.370***
$R^{2}$	0.008	0.554	0.617	0.651	0.668
$\Delta R^{2}$	0.003	0.551	0.613	0.648	0.664
*F*	1.493	180.095**	194.069**	224.969**	207.410**

Variables	Model					
	Social isolation			Professional identification		
	12	13	14	15	16	17
Gender	0.037	0.043	0.020	0.005	−0.001	0.020
Age	0.021	0.010	0.007	−0.079*	−0.069*	−0.067*
Education attainment	0.023	0.030	0.047*	−0.005	−0.012	−0.027
Working hours	−0.060*	−0.022	−0.029	0.038	0.005	0.010
Humble leader behavior		−0.734***	−0.725***		0.660***	0.653***
Relational identification			−0.203***			0.162***
Humble leader behavior *Relational identification			−0.170***			0.159***
$R^{2}$	0.006	0.544	0.616	0.008	0.443	0.495
$\Delta R^{2}$	0.001	0.540	0.612	0.002	0.439	0.490
*F*	1.153	172.713**	165.368**	1.400	115.120**	101.347**

***, **, * represent 1, 5, and 10% significance levels, respectively.

Hypothesis 1b predicted that humble leader behavior has a significant positive effect on employees’ job accomplishment. As shown in Table 5, adding only the control variable in Model 4 and further adding the independent variable humble leader behavior in Model 5 showed that humble leader behavior has a significant positive effect on job accomplishment (*β* = 0.670, *p* < 0.001), and Hypothesis 1b was supported.

Hypothesis 2 predicted that humble leader behavior has a significant negative effect on occupational stigma consciousness. As shown in Table 5, adding only the control variable in Model 7 and further adding the independent variable humble leader behavior in Model 8 showed that humble leader behavior has a significant negative effect on occupational stigma consciousness (*β* = 0.740, *p* < 0.001), and Hypothesis 2 was supported.

Hypothesis 3a predicted that occupational stigma consciousness has a significant negative effect on proactive behavior. As shown in Table 5, adding only the control variable and independent variable humble leader behavior in Model 2 and further adding the independent variable occupational stigma consciousness in Model 3 showed that occupational stigma consciousness has a significant negative effect on proactive behavior (*β* = −0.448, *p* < 0.01), and Hypothesis 3a was supported.

Hypothesis 3b predicted that occupational stigma consciousness has a significant negative effect on job accomplishment. As shown in Table 5, adding only the control variable and independent variable humble leader behavior in Model 5 and further adding the independent variable occupational stigma consciousness in Model 6 showed that occupational stigma consciousness has a significant negative effect on job accomplishment (*β* = −0.346, *p* < 0.001), and Hypothesis 3b was supported.

### 4.4 Mediation effect test

The results of the Stratified regression analysis presented in Table 3 indicate that the mediating effects of social isolation and professional identification have been preliminarily verified. Subsequently, the Bootstrapping method was employed to assess the robustness of the mediating effects and chain mediating effects between social isolation and professional identification, as illustrated in Table 4. This approach served to further substantiate the mediating roles of social isolation and professional identification.

Hypothesis 4a predicted that humble leader behavior has a significant negative effect on social isolation. As shown in Table 6, adding only the control variable in Model 12 and further adding the independent variable humble leader behavior in Model 13 showed that humble leader behavior has a significant negative effect on social isolation (*β* = −0.734, *p* < 0.001), and Hypothesis 4a was supported.

**Table 6:** Bootstrapping test for mediating effects of social isolation and professional identification.

Intermediary variable	Effect category	Effect size	Standard error	95% confidence interval	
				Lower limit	Limit
Social isolation	Total effect	−0.7125	0.0239	−0.7595	−0.6656
	Indirect effect	−0.3292	0.0283	−0.3852	−0.2755
	Direct effect	−0.3834	0.0312	−0.4445	−0.3222
Professional identification	Total effect	−0.7125	0.0239	−0.7595	−006656
	Indirect effect	−0.2124	0.0216	−0.2566	−0.1704
	Direct effect	−0.5002	0.0296	−0.5584	−0.4420

Hypothesis 4b predicted that social isolation has a significant positive effect on occupational stigma consciousness. As shown in Table 6, adding the control variable and independent variable humble leader behavior in Model 8 and further adding the independent variable social isolation in Model 10 showed that social isolation has a significant positive effect on occupational stigma consciousness (β = 0.461, *p* < 0.001), and Hypothesis 4b was supported.

As demonstrated in Model 10, following the inclusion of social isolation, the impact of humble leader behavior on occupational stigma consciousness remains statistically significant. However, the regression coefficient is diminished to (β = −0.402, *p* < 0.001) in comparison with Model 8. This outcome suggests that social isolation plays a partial mediating role between humble leader behavior and occupational stigma consciousness. Therefore, Hypothesis 4 is validated.

Hypothesis 5a predicted that humble leader behavior has a significant positive effect on professional identification. As shown in Table 6, adding only the control variable in Model 15 and further adding the independent variable humble leader behavior in Model 16 showed that humble leader behavior has a significant positive effect on professional identification (*β* = 0.660, *p* < 0.001), and Hypothesis 5a was supported.

Hypothesis 5b predicted that professional identification has a significant negative effect on occupational stigma consciousness. As shown in Table 6, adding the control variable and independent variable humble leader behavior in Model 8 and further adding the independent variable professional identification in Model 9 showed that professional identification has a significant negative effect on occupational stigma consciousness (*β* = −0.335, *p* < 0.001), and Hypothesis 5b was supported.

As demonstrated in Model 9, following the inclusion of professional identification, the impact of humble leader behavior on occupational stigma consciousness remains statistically significant. However, the regression coefficient is diminished to (*β* = −0.518, *p* < 0.001) in comparison with Model 8. This outcome suggests that professional identification plays a partial mediating role between humble leader behavior and occupational stigma consciousness. Therefore, Hypothesis 5 is validated.

In addition, to test the significance of the mediating effect, the present study used the Bootstrapping method of the Process V4.1 macro program in SPSS26.0 to repeat the sampling 5,000 times to estimate the 95% bias-corrected confidence intervals of the mediating effect values, and the mediating effects of social isolation and professional identification were further verified.

The results of the analysis are shown in Table 6, the indirect effect of social isolation between humble leader behavior and occupational stigma consciousness is −0.3292 with a standard error of 0.0283 and a 95% confidence interval of (−0.3285, −0.2755) excluding 0, which indicates that the mediating effect of social isolation between humble leader behavior and occupational stigma consciousness is significant, which further supports hypothesis 4; The indirect effect of professional identification between humble leader behavior and occupational stigma consciousness is −0.2124 with a standard error of 0.0216 and a 95% confidence interval of (−0.2566, −0.1704) excluding 0, which indicates that the mediating effect of professional identification between humble leader behavior and occupational stigma consciousness is significant, which further supports hypothesis 5.

### 4.5 Moderating effects test

In the context of testing interaction terms, the centralization of independent and moderator variables is a methodological strategy employed to mitigate the issue of covariance (Aiken, 1991). To offer a more intuitive visualization of the moderating effect of leadership relational identification, this study further conducted a simple slope test and plotted the moderating effect.

**Figure 3:**
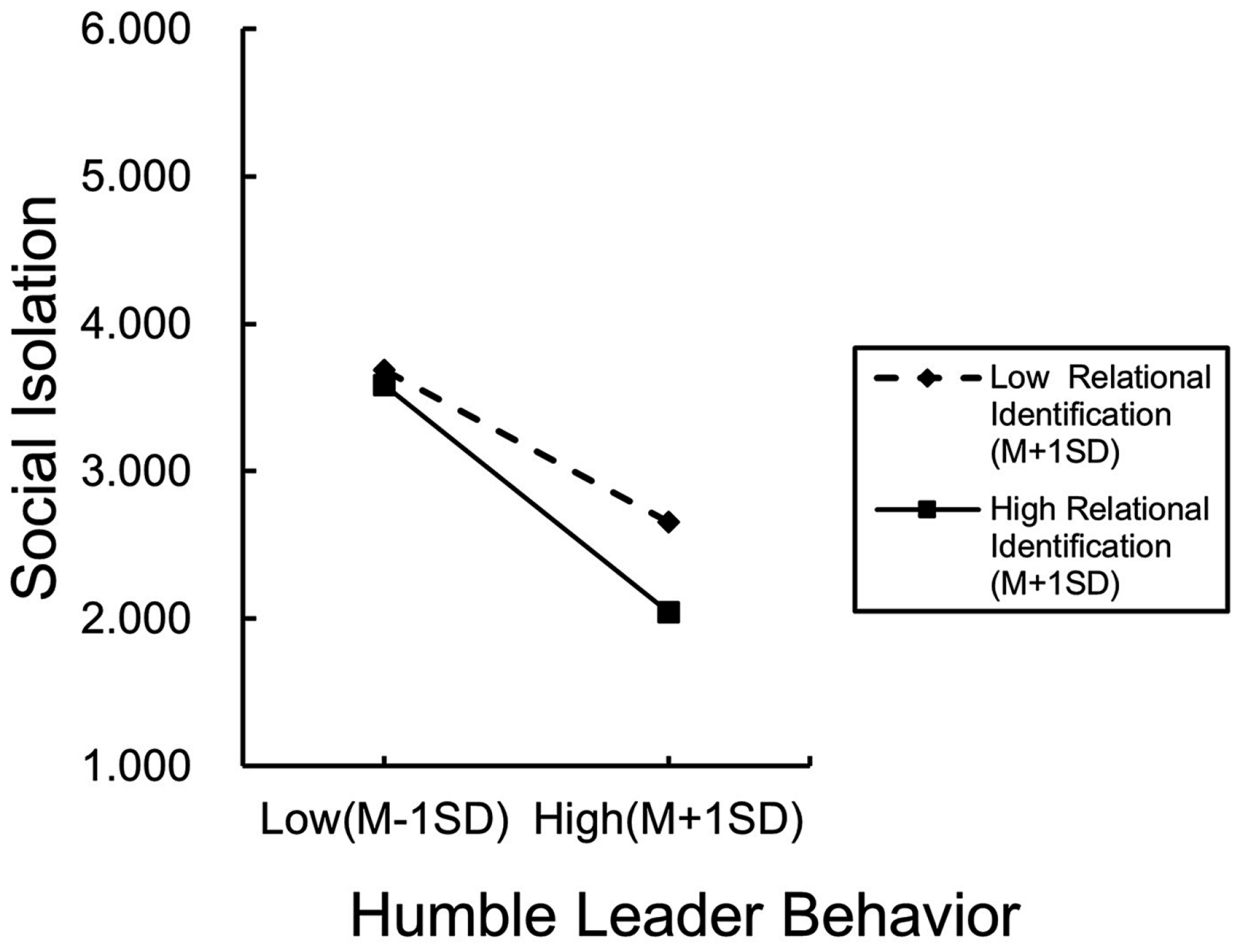
Moderating effect of relational identification on humble leader behavior and social isolation.

Hypothesis 6a proposes that leadership relational identification moderates the negative relation between humble leader behavior and social isolation. Before conducting the stratified regression, the interaction term was constructed after centering on leadership relational identification and humble leader behavior. The results, as shown in Model 14 of Table 5, indicate that the interaction term of leadership relational identification and humble leader behavior has a significant negative effect on social isolation (*β* = −0.170, *p* < 0.001). The results of the simple slope test (Figure 3) indicated that when the level of leadership relational identification was low, humble leader behavior had a significant negative effect on social isolation (*β* = −0.610, *p* < 0.001). Conversely, when the level of leadership relational identification was high, the significant negative effect of humble leader behavior on social isolation increased significantly (*β* = −0.915, *p* < 0.001), thereby supporting Hypothesis 6a.

**Figure 4:**
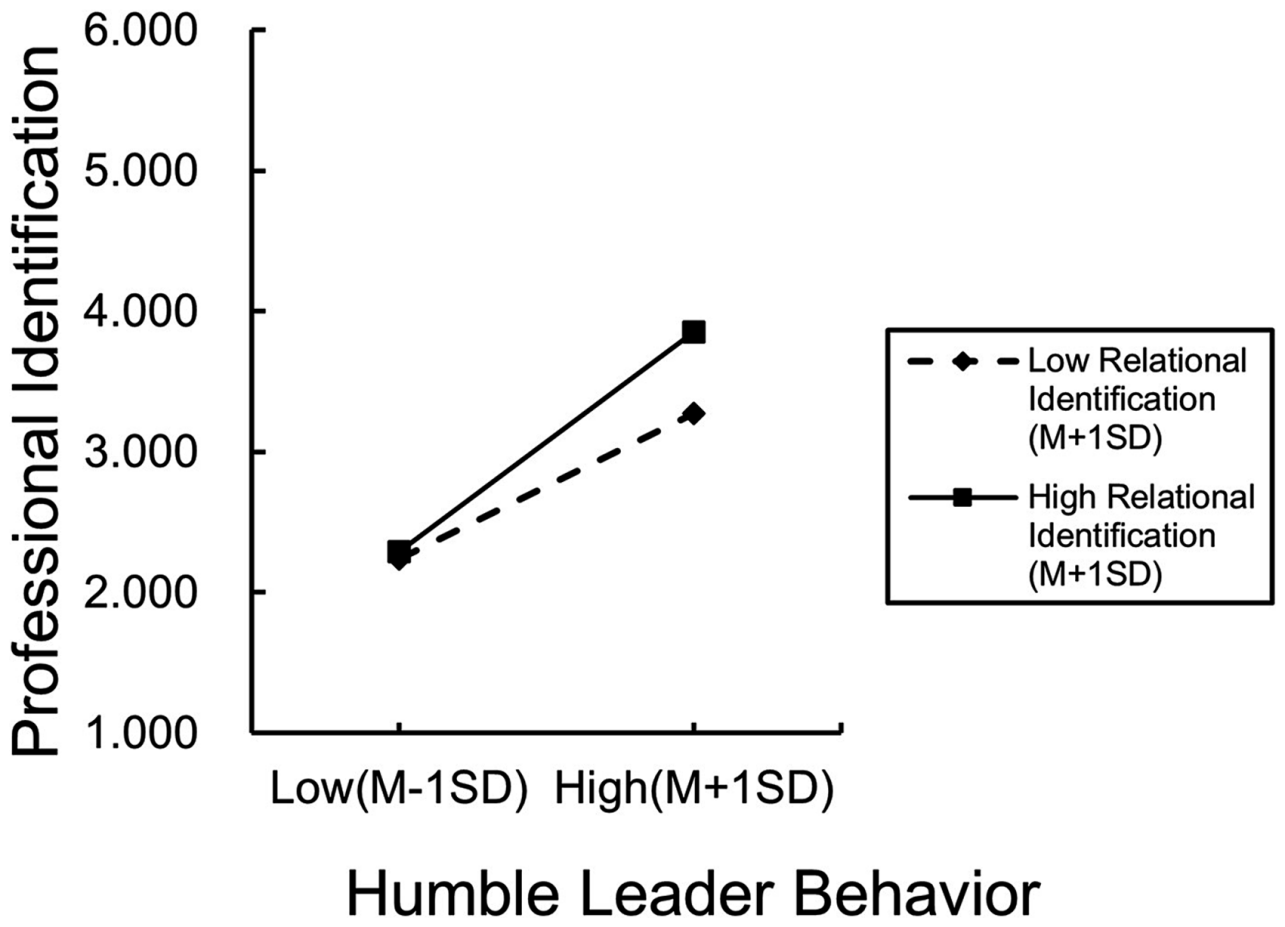
Moderating effect of relational identification on humble leader behavior and professional identification.

Hypothesis 6b proposes that leadership relational identification moderates the positive relation between humble leader behavior and professional identification. Before conducting the stratified regression, the interaction term was constructed after centering on leadership relational identification and humble leader behavior. The results, as shown in Model 17 of Table 5, indicate that the interaction term of leadership relational identification and humble leader behavior has a significant positive effect on professional identification (β = −0.159, *p* < 0.001). The results of the simple slope test (Figure 4) indicated that when the level of leadership relational identification was low, humble leader behavior had a significant positive effect on professional identification (β = 0.614, *p* < 0.001). Conversely, when the level of leadership relational identification was high, the significant positive effect of humble leader behavior on professional identification increased significantly (β = −0.928, *p* < 0.001), thereby supporting Hypothesis 6b.

## 5 Discussion

### 5.1 Theoretical significance

Based on the social exclusion theory and the conservation of resources theory (COR), this study proposes a dual-path model of “social isolation-occupational identity” of occupational stigma, which deepens the understanding of the mechanism of leadership, and the humble leader not only directly alleviates occupational stigma, but also indirectly optimizes the psychological state of the employees by improving their social connection and occupational cognition. In the past, academics have studied the formation mechanism of occupational stigma from two perspectives: at the macro level, it reveals the systematic impact of structural exclusion on practitioners, and the occupational labeling and ethical alienation of non-decent work from society, which reinforces social marginalization (Ashforth and Kreiner, 1999; Hughes, 1962); and at the micro level, studies focuses on the depletion of individual psychological resources due to employ-ees’ occupational stigmatization. It can trigger a professional identity crisis when they have devalued self-worth (Simpson et al., 2012). By promoting teamwork, humble leader behavior can alleviate social isolation and weaken the pressure of external rejection; emphasizing the social value of work and reaffirming the work’s assignment to society makes it easier for employees to reconstruct the meaning of their careers.

The study explores the differentiated intervention paths of humble leader behavior on occupational stigma by constructing a dual-mediation model of “social isolation-occupational identity”: at the external environment level, humble leader behavior strengthen open communication and empowerment decision-making to promote teamwork, weaken the power distance within the organization, and effectively reduce the negative impacts of social isolation, thus alleviating the marginalization predicament of non-decent work practitioners. At the individual psychological level, the humble leader behavior reaffirms the value and cognitive reconstruction of employees, publicly recognizes the contribution of the occupation, emphasizes the social significance of work, and helps employees to reconstruct the value and significance of their occupational identities, and transforms the originally stigmatized occupational labels into identities with a sense of responsibility. This model takes into account the dual paths of social environment optimization and individual psychological empowerment, provides a more comprehensive theoretical basis for the intervention strategy of occupational stigma, and makes up for the limitations of single-perspective research. The study further introduces leadership relationship identity as a moderator variable to deepen the understanding of the boundary conditions of leadership effectiveness. When employees’ leadership relationship identity is high, the behaviors of humble leaders are more likely to be interpreted as sincere support and care, which strengthens professional identity and motivates employees to actively participate in interactions to alleviate social isolation through a two-way feedback mechanism; conversely, if relationship identity is low, the role of humble leaders may be limited. This finding explores the role of the quality of superior-subordinate interactions in the management of occupational stigma, both highlighting the moderating significance of affective connections on the efficacy of leadership behaviors and providing theoretical support for how to optimize management effectiveness through relationship building in organizational practice.

This study revisits the mechanism of occupational stigma management from a resource-gain perspective and proposes that humble leaders can form a virtuous cycle of resource regeneration by integrating social support and psychological empowerment. Unlike previous studies with a static perspective or a single point in time (Schaubroeck et al., 2018), this study uses multi-temporal tracking and structural equation modeling to validate that humble leaders not only directly enhance employees’ proactive behaviors and job achievements, but also indirectly mitigate the negative impact of occupational stigma by reducing social isolation and enhancing occupa-tional identity. The results of this study show that humble leaders alleviate the external pressure of occupational stigma through social resources such as teamwork, and at the same time enhance the internal resilience of individuals through psychological resources such as occupational identity enhancement, to encourage employees to gradually break the stigmatization dilemma in the accumulation of resources. For example, funeral industry leaders transform the originally stigmatized occupational labels into identity symbols with social value by enhancing employees’ occupational dignity and professional self-confidence (Wang et al., 2024). We explored resource conservation theory’s research on the unidirectionality of resource flow and attempted to analyze the possibility that social and psychological resources promote the continuous accumulation of resources through benign interaction. The finding explores a new direction for the study of psychological capital in adversity, emphasizing that organizational interventions can reshape the fitness of the individual and the environment through multiple resource paths.

### 5.2 Practical significance

Leaders should enhance employees’ sense of psychological security through humble leadership behaviors and help them reconstruct their perception of professional values. For example, the organization can strengthen the recognition of professional value through regular recognition activities and the establishment of perfect career promotion channels; at the same time, leaders need to strengthen personalized care and resource support, such as the establishment of career development channels and the provision of mental health resources, to help employees accumulate stigma-resistant capital to avoid burnout exacerbated by the sense of isolation and seriously affected by the sense of occupational stigma; once again, cross-departmental collaborative projects (e.g., “Cleaners and Community Connections” program) are available, can be used to break down the social barriers of occupational groups, promote social identity, and alleviate social isolation. In addition, online mutual support platforms can be set up, and non-decent workers can be encouraged to share their occupational stories and coping experiences in the form of online tree holes. This not only creates a collective narrative of “destigmatization” among employees, but also allows leaders to better understand the sources of stigma pressure among employees and formulate relevant intervention programs, so that management measures can be precise and effective, and provide employees with a warm and united social environment, thus building organizational resilience against stigma and reducing the negative impacts of stigmatization.

Therefore, managers should focus on establishing high-quality leader-member relationships with their employees, enhancing their emotional attachment to their leaders through regular feedback, personalized care, and transparent communication, to increase the credibility and influence of humble behaviors and strengthen the construction of relational identity, to enhance the effectiveness of humble leadership behaviors. For example, in the nurses’ group, a three-tier dialogue mechanism can be set up to ensure that grassroots demands reach the decision-making level. Second, organizations should promote an inclusive culture and reshape social perceptions of non-decent work through anti-discrimination policies and public advocacy. For example, the organization should join hands with the media to plan documentaries or special reports to show the professionalism and social value of non-decent work practitioners. Finally, it is recommended that humble leadership be included in the selection criteria for managers, and that scenario simulation training be used to enhance leaders’ sensitivity to the psychological resources of their employees, to increase the effectiveness of their leadership.

### 5.3 Limitations and future research

Although our paper contributes to the literature, it has some potential limitations. Firstly, the data for the study were mainly derived from the nurses’ group, which has a small number of male staff and an imbalanced gender ratio, which may affect the generalizability of the findings in terms of gender. Although nurses are a typical non-decent work group, there may be differences in the stigmatizing characteristics of other occupations, and the cross-occupational applicability of the study’s findings needs to be further verified. We encourage the future inclusion of more non-decent occupational groups to validate the generalizability of this study’s findings and enhance the external validity of the conclusions. Second, the study is based on the Chinese context, where the role of humble leadership may be influenced by the collectivist culture, and its applicability in the individualistic culture is unclear. Future research could further determine the generalizability or differences of our findings across cultures and contexts, and could compare the perceived occupational stigma of non-decent work and its relationship with leadership styles across cultures, and explore the influence of cultural factors. Third, although we validated the mediating role of social isolation and professional identity and the moderating role of leadership relationship identity, we did not explore in depth the effects of other potential mechanisms on stigma, nor did we examine the synergistic effects of situational factors, and we hope that in the future we will be able to explore the effects of the role of psychological capital in stigma alleviation, or introduce moderating variables such as organizational fairness and team cohesion, construct a more comprehensive theoretical model.

## 6 Conclusion

This study focuses on the problem of occupational stigma of non-decent workers and reveals the mechanisms of the mitigating effect of humble leadership as well as the behavioral impacts and boundary conditions. It was found that humble leaders can significantly reduce employees’ occupational stigma of non-decent work, and this effect is mainly achieved through reducing employees’ social isolation and enhancing employees’ sense of occupational identity. Further research shows that reduced occupational stigma significantly enhances employees’ proactive behaviors and job achievement. It was found that when employees have a high level of relational identification with their leaders, the reduction of social isolation and the enhancement of occupational identity by humble leaders are more significant, thus reducing employees’ occupational stigma more effectively. Specifically, employees with a high level of relational identity are more likely to interpret leadership behaviors as “sincere care” rather than “strategic management,” and thus accept the values conveyed by their leaders more actively, resulting in a stronger professional identity and social connection. This finding suggests that relational identity plays an important borderline role in the influence of humble leaders on employees’ sense of professional stigma.

## Data Availability

The raw data supporting the conclusions of this article will be made available by the authors, without undue reservation.
