# Supplementary material for: When leaders “stoop”: dual-path mechanisms and social connection reconstruction of humble leader behavior to dispel occupational stigma consciousness
Source: Front Psychol. 2025 Sep 29;16:1614345. doi: 10.3389/fpsyg.2025.1614345 (PMC12519094; doi:10.3389/fpsyg.2025.1614345)
Supplement: Supplementary file 1 [file Table_1.docx]

Supplementary Material

# Exploratory factor analysis

During the interviews, a pre-survey was conducted using paper questionnaires on 180 participants from the hospitals where the interviewed nurses were employed. The collected samples were then subjected to exploratory factor analysis (EFA) and reliability testing to eliminate any invalid items, resulting in a total of 180 valid samples. The participants were asked to provide information on their demographic characteristics and were then assessed in terms of their humble behaviour, leadership relationship identification, social isolation and occupational identity. Furthermore, the participants were asked to disclose information regarding any feelings of shame they might have experienced in their professional lives. In addition to these assessments, nurse managers were invited to provide a rating of their nurses' proactive behaviour and professional achievements. The measurement of the data was conducted using a 5-point Likert scale, where 1 represented "strongly disagree" and 5 represented "strongly agree." The demographic variable information table for the sample is shown in Table 1. Initially, exploratory factor analysis was conducted on the data utilising SPSS26.0 software. The KMO measure was determined to be 0.969, with 1,326 degrees of freedom and an approximate chi-square value of 9,294.527, p < 0.001. This indicated that the sample data was suitable for exploratory factor analysis.

Secondly, principal component analysis was employed to extract seven factors, which were subsequently rotated using Kaiser's normalised maximum variance method. The resultant scale comprised seven factors and 52 items. The scale content and loadings are displayed in the following table, with the seven factors collectively accounting for 73.512% of the total variance. The dimensions of the model are as follows: Dimension 1 is Leadership Relationship Identification, comprising 10 items; Dimension 2 is Humble Leadership, comprising 9 items; Dimension 3 is Social Isolation, comprising 6 items; Dimension 4 is Occupational Identification, comprising 3 items; Dimension 5 is Occupational Anonymity, comprising 10 items; Dimension 6 is Work Achievement, comprising 5 items; and Dimension 7 is Proactive Behaviour, comprising 9 items.

In addition, a reliability assessment was conducted. The overall Cronbach's α for the 52 items in question was found to be 0.846. More specifically, the α for leadership relationship identification was 0.862, the α for humble leadership was 0.884, and the α for social isolation was 0.As demonstrated in Table 8.22, the Cronbach's alpha for occupational identity was 0.866, the Cronbach's alpha for occupational stigma was 0.812, the Cronbach's alpha for work achievement was 0.875, and the Cronbach's alpha for employee proactivity was 0.852. The scale demonstrated a satisfactory degree of reliability.

**Table 1.** KMO and Bartlett tests

| **KMO test** | | 0.969 |
| --- | --- | --- |
| **Bartlett sphericity test** | **Approximate chi-square** | 9294.527 |
|  | **Degree of freedom** | 1326 |
|  | **Significance** | 0.000 |

**Table 2.**  The results of the exploratory factor analysis

|  | **RI** | **HLB** | **SI** | **PI** | **OSC** | **JA** | **PB** |
| --- | --- | --- | --- | --- | --- | --- | --- |
| **RI 1** | *0.862* | 0.277 | 0.050 | -0.209 | -0.061 | -0.092 | 0.129 |
| **RI 2** | *0.864* | 0.451 | -0.010 | -0.027 | -0.153 | 0.080 | 0.035 |
| **RI 3** | *0.890* | 0.510 | -0.074 | 0.021 | 0.043 | 0.040 | -0.017 |
| **RI 4** | *0.828* | 0.516 | 0.200 | 0.060 | 0.074 | 0.001 | 0.074 |
| **RI 5** | *0.885* | 0.454 | 0.090 | 0.011 | 0.067 | -0.197 | 0.113 |
| **RI 6** | *0.815* | 0.357 | 0.053 | -0.006 | 0.054 | 0.245 | -0.217 |
| **RI 7** | *0.822* | 0.304 | -0.039 | 0.155 | -0.040 | -0.018 | -0.385 |
| **RI 8** | *0.889* | 0.309 | 0.007 | 0.107 | -0.051 | 0.100 | -0.097 |
| **RI 9** | *0.894* | 0.336 | 0.308 | -0.042 | -0.079 | -0.010 | 0.080 |
| **RI 10** | *0.810* | 0.305 | 0.042 | 0.085 | 0.303 | -0.228 | 0.015 |
| **HLB 1** | 0.642 | *0.859* | 0.020 | 0.408 | 0.327 | 0.091 | 0.034 |
| **HLB 2** | 0.562 | *0.893* | -0.060 | 0.065 | 0.513 | 0.306 | 0.030 |
| **HLB 3** | 0.679 | *0.863* | -0.015 | 0.204 | -0.247 | -0.013 | 0.232 |
| **HLB 4** | 0.579 | *0.891* | 0.026 | 0.034 | 0.199 | 0.125 | 0.552 |
| **HLB 5** | 0.601 | *0.879* | 0.113 | 0.444 | 0.148 | 0.018 | -0.217 |
| **HLB 6** | 0.532 | *0.863* | 0.015 | 0.121 | 0.099 | 0.637 | 0.061 |
| **HLB 7** | 0.594 | *0.881* | 0.168 | 0.219 | -0.017 | 0.310 | 0.108 |
| **HLB 8** | 0.524 | *0.847* | 0.131 | 0.076 | 0.667 | -0.012 | 0.106 |
| **HLB 9** | 0.525 | *0.802* | 0.132 | 0.658 | 0.024 | 0.117 | 0.058 |
| **SI 1** | -0.775 | -0.272 | *0.809* | -0.156 | -0.062 | -0.046 | -0.069 |
| **SI 2** | -0.643 | -0.393 | *0.802* | -0.024 | -0.052 | -0.244 | -0.059 |
| **SI 3** | -0.852 | -0.142 | *0.895* | -0.145 | -0.084 | -0.089 | -0.047 |
| **SI 4** | -0.735 | -0.254 | *0.863* | -0.223 | -0.200 | -0.163 | -0.097 |
| **SI 5** | -0.733 | -0.284 | *0.876* | -0.118 | -0.109 | -0.193 | -0.187 |
| **SI 6** | -0.813 | -0.163 | *0.860* | -0.223 | -0.033 | -0.088 | -0.124 |
| **PI 1** | 0.644 | 0.142 | 0.103 | *0.889* | 0.077 | 0.089 | 0.186 |
| **PI 2** | 0.617 | 0.384 | 0.181 | *0.860* | 0.197 | 0.105 | 0.076 |
| **PI 3** | 0.648 | 0.257 | 0.042 | *0.869* | 0.082 | -0.083 | 0.214 |
| **OSC 1** | -0.843 | -0.115 | -0.109 | 0.056 | *0.806* | 0.003 | 0.075 |
| **OSC 2** | -0.899 | -0.102 | 0.018 | -0.036 | *0.889* | -0.085 | 0.073 |
| **OSC 3** | -0.789 | -0.276 | -0.203 | 0.065 | *0.827* | -0.086 | 0.068 |
| **OSC 4** | -0.860 | -0.131 | -0.027 | 0.020 | *0.862* | -0.163 | -0.021 |
| **OSC 5** | -0.819 | -0.186 | -0.081 | 0.046 | *0.874* | 0.003 | 0.173 |
| **OSC 6** | -0.862 | -0.173 | -0.015 | -0.109 | *0.865* | -0.033 | 0.028 |
| **OSC 7** | -0.803 | -0.177 | -0.194 | 0.094 | *0.803* | -0.159 | 0.178 |
| **OSC 8** | -0.859 | -0.220 | -0.020 | -0.019 | *0.808* | -0.110 | 0.006 |
| **OSC 9** | -0.811 | -0.232 | -0.099 | 0.077 | *0.843* | -0.107 | 0.053 |
| **OSC 10** | -0.861 | -0.187 | -0.024 | -0.084 | *0.874* | -0.068 | -0.046 |

Continue writing the table from the previous text

|  | RI | HLB | SI | PI | OSC | JA | PB |
| --- | --- | --- | --- | --- | --- | --- | --- |
| **JA 1** | 0.340 | 0.184 | 0.136 | 0.165 | 0.059 | *0.887* | 0.131 |
| **JA 2** | 0.524 | 0.173 | 0.418 | 0.158 | 0.058 | *0.854* | 0.078 |
| **JA 3** | 0.452 | 0.186 | 0.122 | 0.172 | 0.134 | *0.836* | 0.031 |
| **JA 4** | 0.581 | 0.349 | 0.537 | 0.147 | 0.033 | *0.840* | -0.013 |
| **JA 5** | 0.557 | 0.241 | 0.270 | 0.108 | 0.079 | *0.878* | 0.041 |
| **PB 1** | 0.468 | 0.162 | 0.376 | 0.063 | 0.029 | -0.039 | *0.892* |
| **PB 2** | 0.456 | 0.180 | -0.006 | 0.217 | 0.057 | -0.031 | *0.853* |
| **PB 3** | 0.344 | 0.316 | 0.336 | 0.171 | 0.001 | -0.071 | *0.862* |
| **PB 4** | 0.441 | 0.169 | 0.068 | 0.185 | 0.170 | 0.031 | *0.855* |
| **PB 5** | 0.553 | 0.223 | 0.415 | 0.099 | 0.070 | 0.078 | *0.857* |
| **PB 6** | 0.611 | 0.278 | 0.406 | 0.135 | 0.201 | 0.140 | *0.872* |
| **PB 7** | 0.412 | 0.089 | 0.256 | 0.190 | 0.116 | 0.009 | *0.812* |
| **PB 8** | 0.565 | 0.217 | 0.277 | 0.202 | 0.112 | 0.040 | *0.830* |
| **PB 9** | 0.630 | 0.348 | 0.478 | 0.019 | 0.066 | -0.060 | *0.809* |

**Note(s):** RI=Relationship Identification; HLB=Humble Leader Behavior; SI=Social Isolation; PI=Professional Identification; OSC=Occupational Stigma Consciousness; JA=Job Accomplishment; PB=Proactive Behavior； the data presented in italic fonts refer to item loadings of the corresponding construct
